# Tissue Factor Expression Is Associated with Distinct Pattern of Immune-Cell Infiltration in the Ovarian Tumor Microenvironment

**DOI:** 10.3390/ijms27209148

**Published:** 2026-10-15

**Authors:** T. M. Mohiuddin, Fatema Binte Hafiz, Wenjie Sheng, Chaoyu Zhang, Marwah Al-Rawe, Felix Zeppernick, Svetlana Tchaikovski, Ivo Meinhold-Heerlein, Ahmad Fawzi Hussain

**Affiliations:** 1 Department of Gynecology and Obstetrics, Medical Faculty, Justus-Liebig-University Giessen, Klinikstr. 33, 35392 Giessen, Germany; 2 Clinic for Gynecology and Obstetrics, University Hospital Brandenburg, Medizinische Hochschule Brandenburg, Hochstraße 29, 14770 Brandenburg an der Havel, Germany; 3 Institute of Biology, Martin Luther University, Weinbergweg 10, 06120 Halle, Germany

**Keywords:** tissue factor, ovarian cancer, tumor immune microenvironment, immune infiltration, CD8+ T cells, natural killer cells, macrophages

## Abstract

Tissue factor (TF) has been implicated in tumor progression and immune regulation, but its cellular sources and relationship with immune infiltration in ovarian cancer remain incompletely defined. We integrated single-cell RNA sequencing (GSE232314), analyses of The Cancer Genome Atlas (TCGA) ovarian cancer cohort, and multiplex immunofluorescence in an independent 25-case tissue microarray (TMA). Single-cell analysis showed that TF expression was concentrated in tumor epithelial cells and a discrete endothelial subpopulation, with minimal expression in immune and other stromal compartments. In the TCGA cohort, TF expression was not significantly associated with tumor stage, grade, disease-free survival, or overall survival. In TF-stratified analyses, however, high CD8^+^ T cell infiltration was associated with improved survival in the low-TF expression subgroup (HR = 0.60) but with worse survival in the high-TF subgroup (HR = 1.72); high M1 macrophage infiltration was associated with improved survival only in the low-TF subgroup. TF expression correlated negatively with estimated CD8^+^ T cell, memory B cell, and M1 macrophage infiltration and positively with naïve B cell infiltration. In the TMA cohort, high-TF tumors showed significantly lower CD56^+^ NK-cell and CD68^+^ macrophage infiltration and a lower CD68/CD163 ratio. Together, these findings show cross-sectional associations between TF expression and reduced measured abundance of several antitumor immune populations and a stratum-dependent association between CD8^+^ T cell infiltration and survival. These associations were not fully concordant between the transcriptomic (TCGA) and protein-level (TMA) cohorts and require prospective, formally tested validation. TF warrants further investigation as a candidate immune-context biomarker in ovarian cancer.

## 1. Introduction

Ovarian cancer is one of the most lethal gynaecologic malignancies and remains a major cause of cancer-related death among women worldwide [1]. Despite advances in surgical management and platinum-based chemotherapy, long-term outcomes remain poor, largely because many patients present with advanced-stage disease and subsequently develop recurrent, treatment-resistant tumors [2,3]. High-grade serous ovarian carcinoma (HGSOC), the most common histologic subtype, is characterized by marked molecular heterogeneity and a complex tumor microenvironment that contributes to disease progression and therapeutic resistance [4,5]. Identifying biomarkers that capture both tumor-intrinsic biology and immune regulation may therefore improve prognostic stratification and guide the development of new therapeutic strategies.

Tissue factor (TF), a transmembrane glycoprotein, is the principal initiator of the extrinsic coagulation cascade [6,7]. Beyond its established role in hemostasis, accumulating evidence implicates TF in angiogenesis, tumor growth, metastasis, and inflammation [8,9,10]. Aberrant TF expression has been reported in pancreatic, breast, colorectal, lung, ovarian, and other cancers [11,12,13,14,15], where it is often associated with aggressive tumor behaviour and adverse clinical outcomes. Through interactions with coagulation factors and protease-activated receptors (PARs), TF-dependent signalling can activate pathways involved in cell proliferation, migration, inflammation, and immune regulation [16,17]. Thus, TF may function not only as a coagulation-associated molecule but also as an important regulator of the tumor microenvironment [18,19].

In ovarian cancer, TF levels tend to be higher when the disease is more severe. Patients with high TF in their blood before surgery tend to survive for less time than those with low TF [20]. High TF in the tumor is also linked to a greater risk of blood clots, especially in the clear-cell type of ovarian cancer [21,22]. Ovarian cancer cells also make their own TF and factor VII, and this pairing seems to help the tumor grow [23], and TF has been proposed as a candidate immunotherapeutic target in this disease [24].

TF overexpression helps tumors evade immune surveillance and is repeatedly linked to a “cold,” immunosuppressive microenvironment across cancer types. In triple-negative breast cancer, tumor-intrinsic TF suppresses chemokines (CXCL9/10/11) needed to recruit T cells, and its loss increases effector T cell infiltration while reducing Tregs [19,25]. Downstream of coagulation, TF-driven thrombin signaling through PAR1 drives CD8+ T cell exhaustion in pancreatic cancer, partly via TREX1-mediated suppression of STING/interferon signaling, and TF-thrombin-generated C5a recruits myeloid-derived suppressor cells and skews T cells toward Treg/Th2 phenotypes [26,27,28,29]. TF expression also correlates negatively with M1-like macrophage and dendritic-cell infiltration and positively with M2-like polarization, an effect reinforced through FXa-PAR2 signaling, TF-bearing extracellular vesicles, and TF–FVII-guided macrophage migration along fibrin, and this TF-driven M2 shift has been linked to tumor invasion and metastasis in gastric and lung cancer [30,31,32]. Separately, TF-induced fibrin deposition and platelet activation physically restrict NK-cell infiltration and, via platelet-derived TGF-β, impair NK-cell function [33,34].

The ovarian tumor microenvironment is a dynamic ecosystem of tumor cells, fibroblasts, endothelial cells, immune cells, soluble mediators, and extracellular matrix components that critically influences disease progression and treatment response [35,36]. Bidirectional interactions between tumor cells and infiltrating immune populations contribute significantly to ovarian cancer progression, metastasis, and immune evasion [37]. CD8^+^ T cells, macrophages, natural killer (NK) cells, and B cells are among the immune populations associated with prognosis and therapeutic responsiveness [38,39,40,41,42,43]. In particular, increased infiltration of cytotoxic CD8^+^ T cell infiltration is generally associated with favourable outcomes, whereas tumor-associated macrophages can exert anti-tumor or pro-tumor functions depending on their phenotype, activation state, and spatial context [44,45].

Recent advances in multiplex immunofluorescence and related spatial profiling methods have enabled simultaneous assessment of multiple tumor and immune markers, providing detailed information on cellular heterogeneity, cellular interactions, and tissue organization [46,47,48]. In our previous studies, we used multiplex immunofluorescence to characterize cancer-associated markers in ovarian and breast cancer [12,15,49]. In parallel, single-cell transcriptomics has enabled high-resolution analysis of cellular heterogeneity and immune cell states within ovarian cancers [50,51]. Growing evidence also links coagulation signalling to tumor-associated immune responses [52,53,54]. TF-dependent activation of coagulation proteases and PARs can induce cytokines, chemokines, and proangiogenic mediators within the tumor microenvironment. TF-associated signalling has also been implicated in endothelial dysfunction, leukocyte recruitment, macrophage polarization, and altered NK-cell infiltration or localization in several malignancies [18,31,34]. Ovarian cancer is frequently accompanied by coagulation activation and thrombotic complications, which are associated with advanced disease progression and poor clinical outcomes [55,56]. Although TF is being investigated as a therapeutic target in multiple cancers [57,58], its principal cellular sources in the ovarian tumor microenvironment and its relationship with antitumor immune infiltration remain incompletely defined. It is also unclear whether TF expression is directly associated with survival or instead identifies an immune context in which the prognostic significance of immune infiltration differs.

Accordingly, we integrated single-cell transcriptomic analysis, TCGA-based clinicopathologic and survival analysis, CIBERSORT immune deconvolution, and independent TMA assessment by multiplex immunofluorescence. We aimed to define the cellular distribution of TF expression in ovarian cancer and evaluate its associations with clinicopathologic features, survival, and immune-cell infiltration. This multimodal approach was designed to clarify the relationship between TF and the ovarian tumor immune landscape and to assess the rationale for further investigation of TF as an immune-related biomarker and therapeutic target.

## 2. Results

### 2.1. Single-Cell Transcriptomic Profiling Identifies Tumor Epithelial and Endothelial Cells as the Principal Cellular Sources of TF Expression in Ovarian Cancer

**Figure 1:**
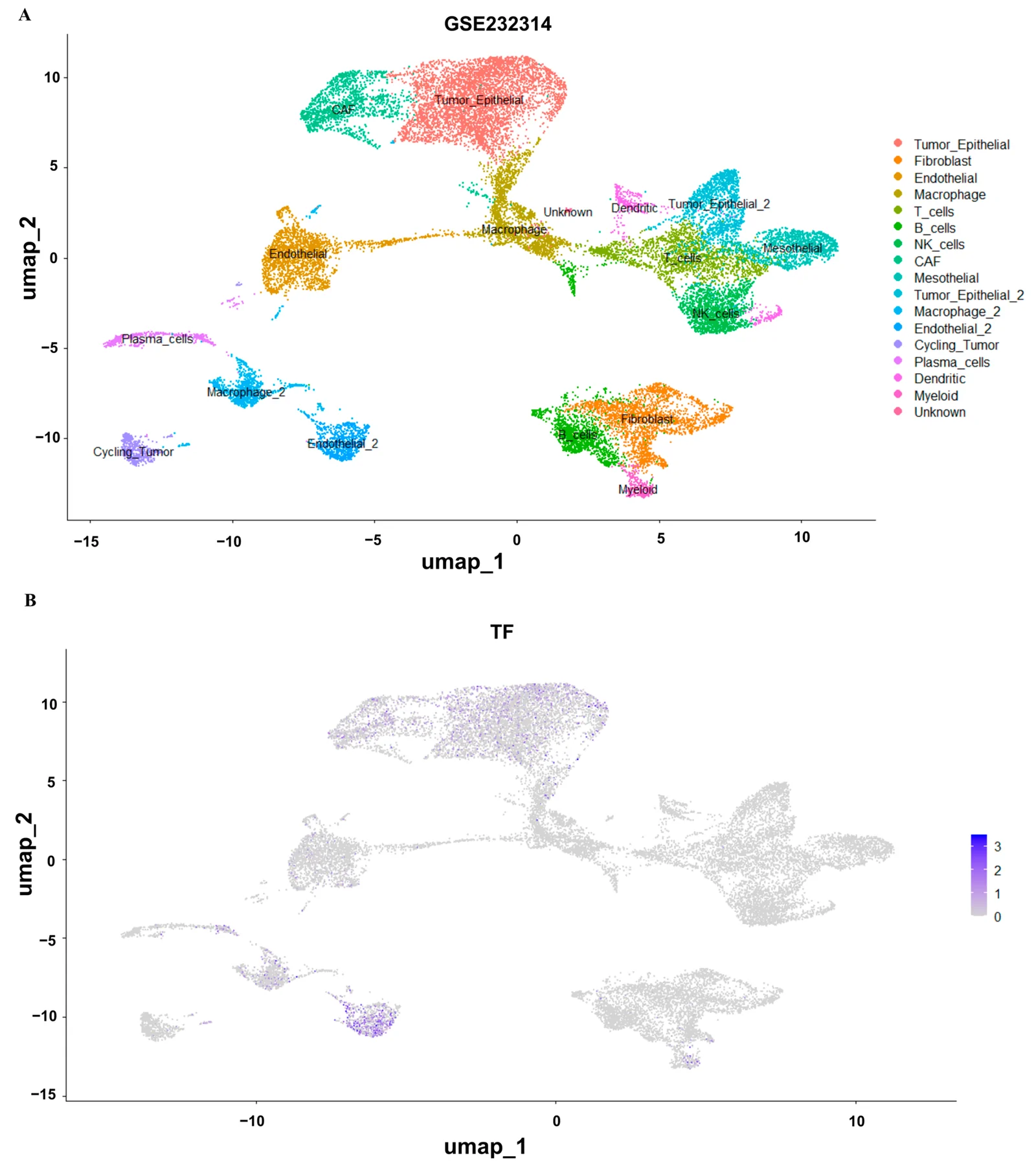
Single-cell transcriptomic localization of tissue factor expression in the ovarian tumor microenvironment. (**A**) UMAP plot showing annotated cell populations identified by graph-based clustering of the GSE232314 single-cell RNA-seq dataset, including tumor epithelial cells (two subclusters), fibroblasts, endothelial cells (two subclusters), macrophages (two subclusters), T cells, B cells, NK cells, cancer-associated fibroblasts, mesothelial cells, dendritic cells, plasma cells, cycling tumor cells, and myeloid cells. (**B**) Feature plot showing TF expression enrichment within tumor epithelial and Endothelial_2 subclusters relative to other populations.

To identify the cell populations expressing TF in the ovarian tumor microenvironment, we analyzed scRNA-seq data from the GSE232314. Uniform manifold approximation and projection (UMAP) clustering resolved distinct populations of tumor epithelial cells (two subclusters), fibroblasts, endothelial cells (two subclusters), macrophages (two subclusters), T cells, B cells, NK cells, cancer-associated fibroblasts, mesothelial cells, dendritic cells, plasma cells, cycling tumor cells, and other myeloid cells (Figure 1A). TF expression was concentrated in tumor epithelial cell clusters and, in the Endothelial_2 subcluster, with minimal expression in the remaining stromal and immune populations (Figure 1B). Genes associated with the TF-high state were enriched for ribonucleoprotein-complex and ribosome biogenesis, rRNA processing, and DNA-replication pathways (Figure S1). Gene set enrichment analysis (GSEA) additionally identified enrichment of MHC class II antigen-presentation and RNA-splicing pathways (Figure S2).

### 2.2. TF Expression Is Not Significantly Associated with Stage, Grade, or Survival in the TCGA Ovarian Cancer Cohort

**Figure 2:**
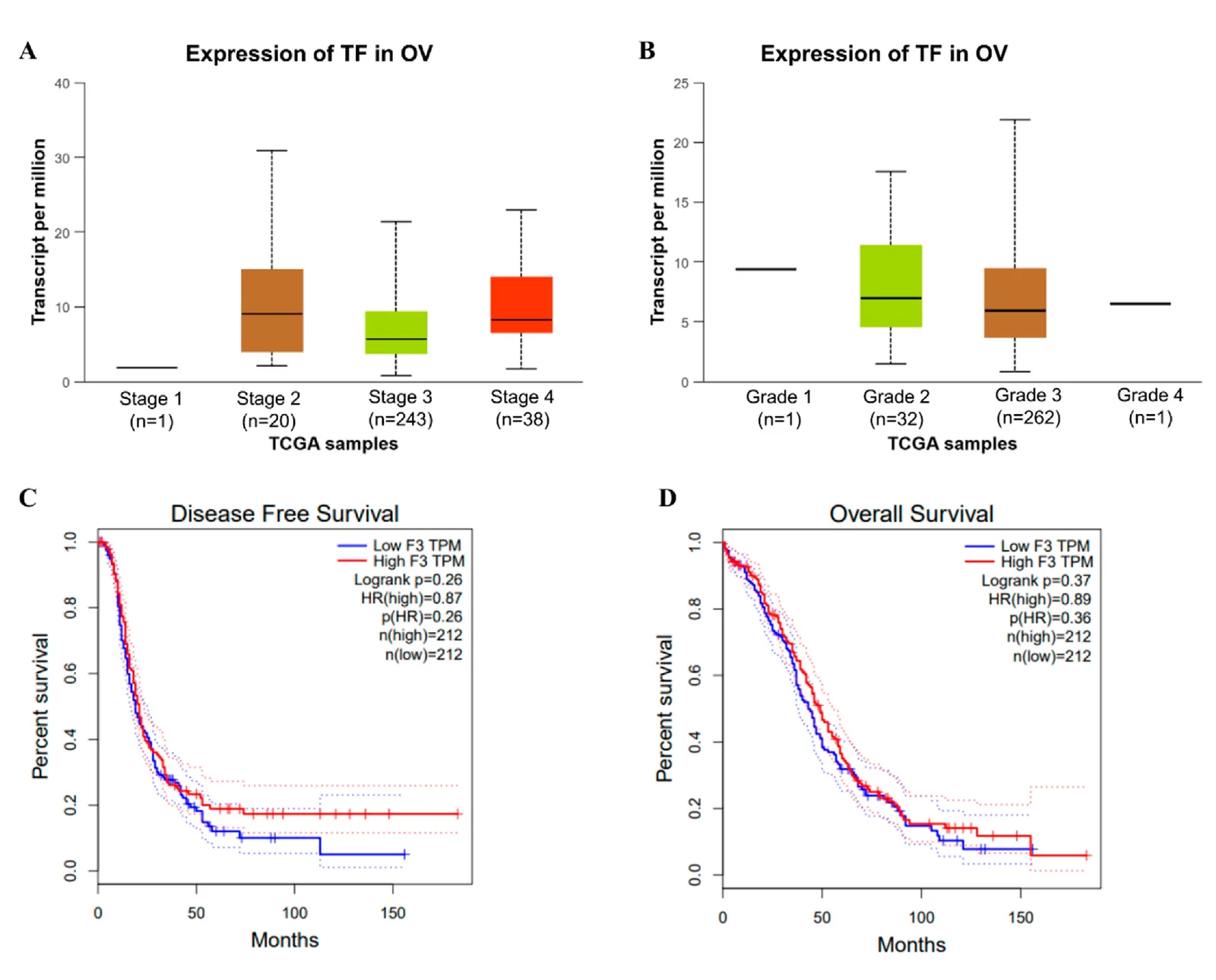
Tissue factor expression and survival outcomes in the TCGA ovarian cancer cohort. (**A**) TF transcript expression (TPM) stratified by pathological tumor stage (Stage I–IV); no statistically significant difference detected (Kruskal-Wallis test, *p* > 0.05). (**B**) TF expression stratified by tumor grade; no statistically significant difference detected (*p* > 0.05). (**C**) Kaplan–Meier disease-free survival curves comparing low- versus high-TF expression groups (n = 212 per group), dichotomized at the median; log-rank *p* = 0.26, HR (high vs. low) = 0.87. (**D**) Kaplan–Meier overall survival curves comparing low- versus high-TF expression groups (n = 212 per group); log-rank *p* = 0.37, HR = 0.89.

TF expression varied across tumor stages and histologic grades, but no statistically significant differences were detected (Figure 2A,B). Median-dichotomized TF expression was also not significantly associated with DFS (log-rank *p* = 0.26, HR for high vs. low TF = 0.87) or OS (log-rank *p* = 0.37; HR = 0.89) (Figure 2C,D). Thus, TF expression alone was not associated with conventional clinicopathological variables or survival in this unadjusted TCGA analysis.

### 2.3. TF-Stratified Analyses Reveal Different Prognostic Associations for CD8+ T Cell and M1 Macrophage Infiltration in Ovarian Cancer

**Figure 3:**
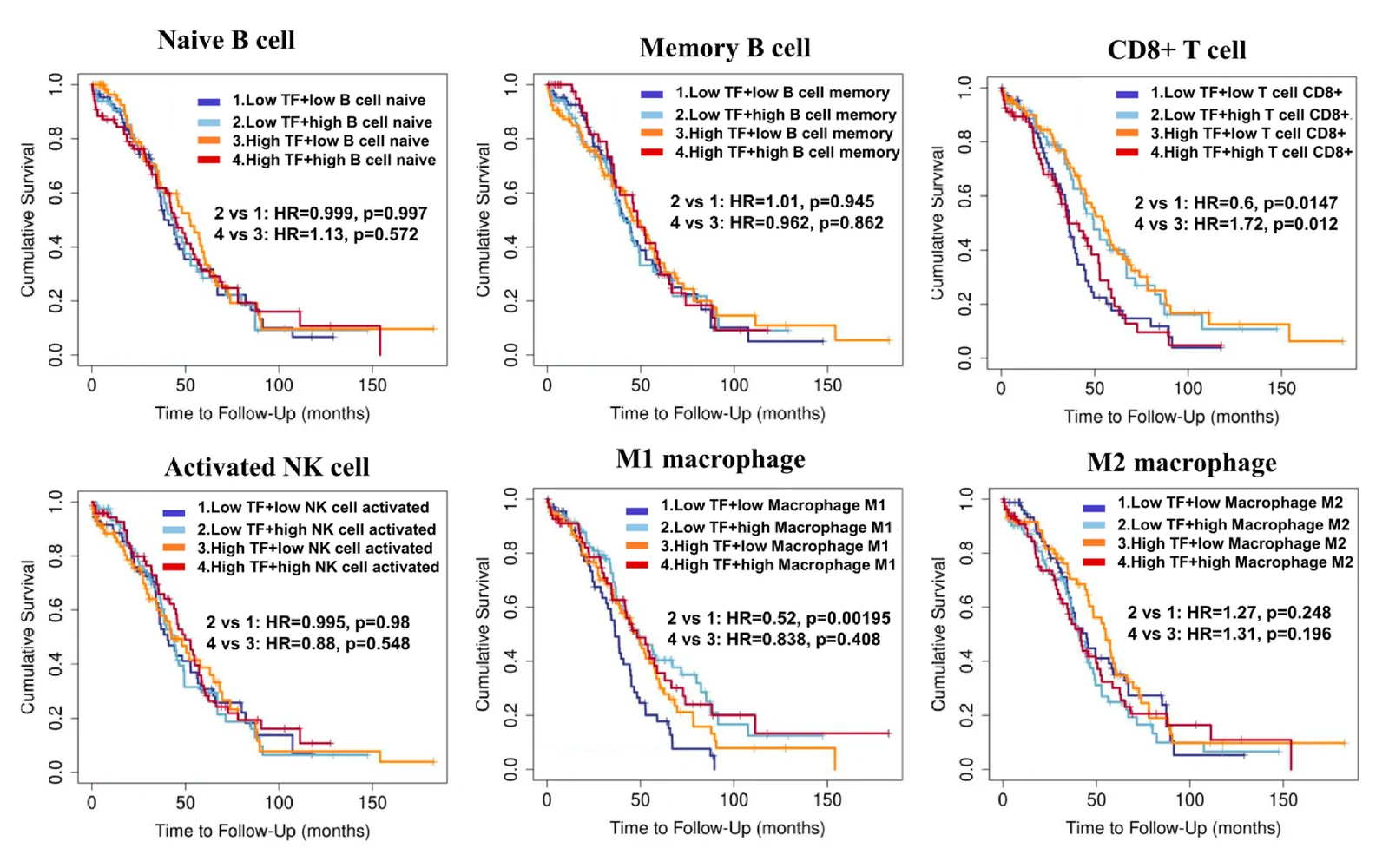
TF-stratified prognostic associations of immune-cell infiltration in ovarian cancer. Kaplan–Meier survival curves for patients categorized into four groups according to TF expression (high/low, median split) and CIBERSORT-derived immune-cell infiltration (high/low, median split). Hazard ratios (HRs) and log-rank *p*-values compare high versus low infiltration within each TF stratum. These are exploratory stratum-specific comparisons and not formal interaction tests.

To examine TF expression in the context of the immune microenvironment, patients were stratified jointly by expression and CIBERSORT-estimated immune-cell abundance (Figure 3). No significant within-stratum survival associations were observed for naïve B cells, memory B cells, or activated NK cells (all *p* > 0.05). For CD8+ T cells, however, high infiltration was associated with better survival in the low-TF subgroup (HR = 0.6, *p* = 0.0147) but with worse survival in the high-TF subgroup (HR = 1.72, *p* = 0.012). High M1 macrophage infiltration was likewise associated with better survival in the low-TF subgroup (HR = 0.52, *p* = 0.00195), whereas this association was not evident in the high-TF subgroup (HR = 0.838, *p* = 0.408). M2 macrophage infiltration showed nonsignificant trends toward poorer survival in both TF strata (low TF: HR = 1.27, *p* = 0.248; high TF: HR = 1.31, *p* = 0.196). These twelve within-stratum comparisons were not corrected for multiple testing; the CD8+ T cell and M1 macrophage findings should therefore be interpreted as exploratory rather than confirmed at a family-wise error rate of 0.05.

### 2.4. TF Expression Is Associated with Selective Differences in Estimated Immune Cell Infiltration in the TCGA Ovarian Cancer Cohort

**Figure 4:**
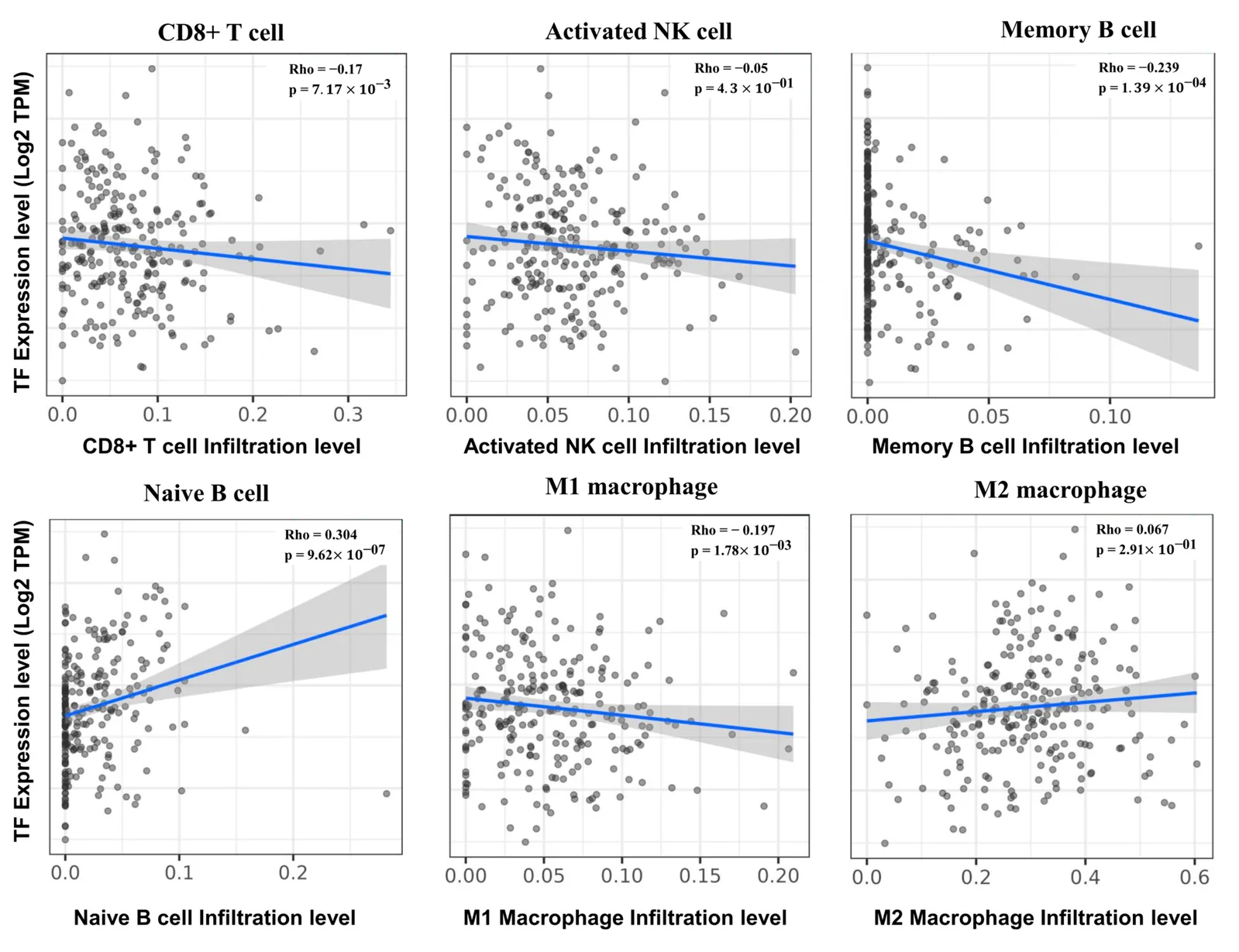
Correlation between tissue factor expression and CIBERSORT-derived immune cell infiltration in the TCGA ovarian cancer cohort. Spearman correlation plots showing the relationship between TF expression and naive B cell, memory B cell, CD8+ T cell, activated NK cell, M1 macrophage, and M2 macrophage infiltration. Blue line represents the fitted trend line, and the grey shaded area represents the 95% confidence interval (CI) around that line.

Spearman correlation analysis of CIBERSORT estimates showed that TF expression was weakly negatively correlated with CD8+ T cell infiltration (ρ = −0.17, *p* = 7.17 × 10^−3^), memory B cell infiltration (ρ = −0.239, *p* = 1.39 × 10^−4^), and M1 macrophage infiltration (ρ = −0.197, *p* = 1.78 × 10^−3^) (Figure 4). TF expression was positively correlated with naïve B cell infiltration (ρ = 0.304, *p* = 9.62 × 10^−7^). No significant correlations were detected for activated NK cells (ρ = −0.05, *p* = 0.43) or M2 macrophages (ρ = 0.067, *p* = 0.291). These findings indicate that TF expression is associated with selective, generally modest differences in the estimated abundance of specific immune-cell populations. We correlated TF expression with key immune checkpoint genes in the TCGA-OV cohort. TF expression correlated positively and significantly with three immune checkpoint genes in the TCGA-OV cohort: PD-L1 (R = 0.15, *p* = 0.0026), CTLA4 (R = 0.15, *p* = 0.0018), and TIM-3 (R = 0.25, *p* = 1.5 × 10^−7^), with the TF-TIM-3 correlation being the strongest and most significant of the three (Figure S3). Correlations with the six CIBERSORT-estimated immune populations and the three immune checkpoint genes were not adjusted for multiple comparisons; the reported *p*-values should be interpreted as exploratory rather than confirmatory.

### 2.5. TMA Analysis Confirms Heterogeneous TF Without Significant Associations with Tumor Stage or Grade

The TMA cohort comprised 25 ovarian carcinoma cases: 22 serous adenocarcinomas, 2 mucinous adenocarcinomas, and 1 endometrioid adenocarcinoma. Stage information was available for 22 cases, including 3 stage I, 9 stage II, and 10 stage III tumors. All 25 cases had grade information: 8 were grade 2, and 17 were grade 3 (Table 1).

**Figure 5:**
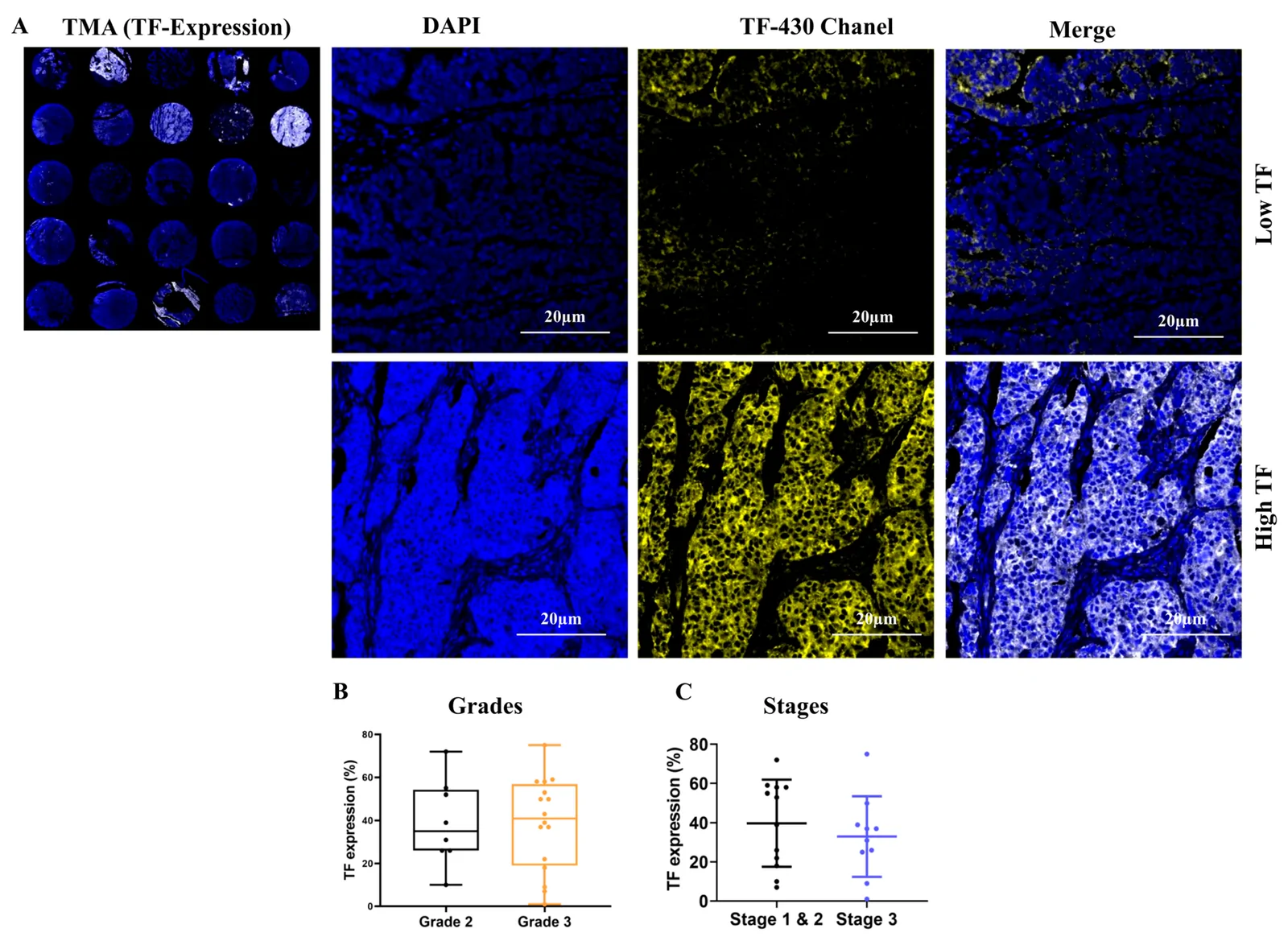
Multiplex immunofluorescence assessment of TF protein expression in an ovarian cancer TMA. (**A**) Representative DAPI, TF-430, and merged images from low-TF and high-TF tumor cores. (**B**) TF-positive cells (%) by tumor grade (grade 2 vs. grade 3); no significant difference was detected (two-tailed Mann-Whitney U test, *p* > 0.05). (**C**) TF-positive cells (%) by tumor stage (stage I–II vs. stage III); no significant difference was detected (*p* > 0.05). Data are shown as mean ± SD with individual sample values overlaid. Scale bar 20 µm.

Multiplex immunofluorescence demonstrated heterogeneous TF protein expression across the ovarian cancer TMA (Figure 5). TF positivity did not differ significantly between grade 2 and grade 3 tumors (Figure 5B). Grade 3 tumors nevertheless showed a broader range of TF-expression values, including TF-negative and markedly TF-high cases. TF expression also did not differ significantly between stage I–II and stage III tumors (Figure 5C); mean TF positivity was 39.9% in stage I-II tumors and 33.3% in stage III tumors, with substantial overlap between groups. These protein-level findings are consistent with the TCGA analyses and indicate that, in this small cohort, TF expression was heterogeneous but not significantly associated with histologic grade or stage.

### 2.6. Multiplex Immunofluorescence Reveals Heterogeneous Spatial Distributions of TF and Immune-Cell Markers in Ovarian Cancer

**Figure 6:**
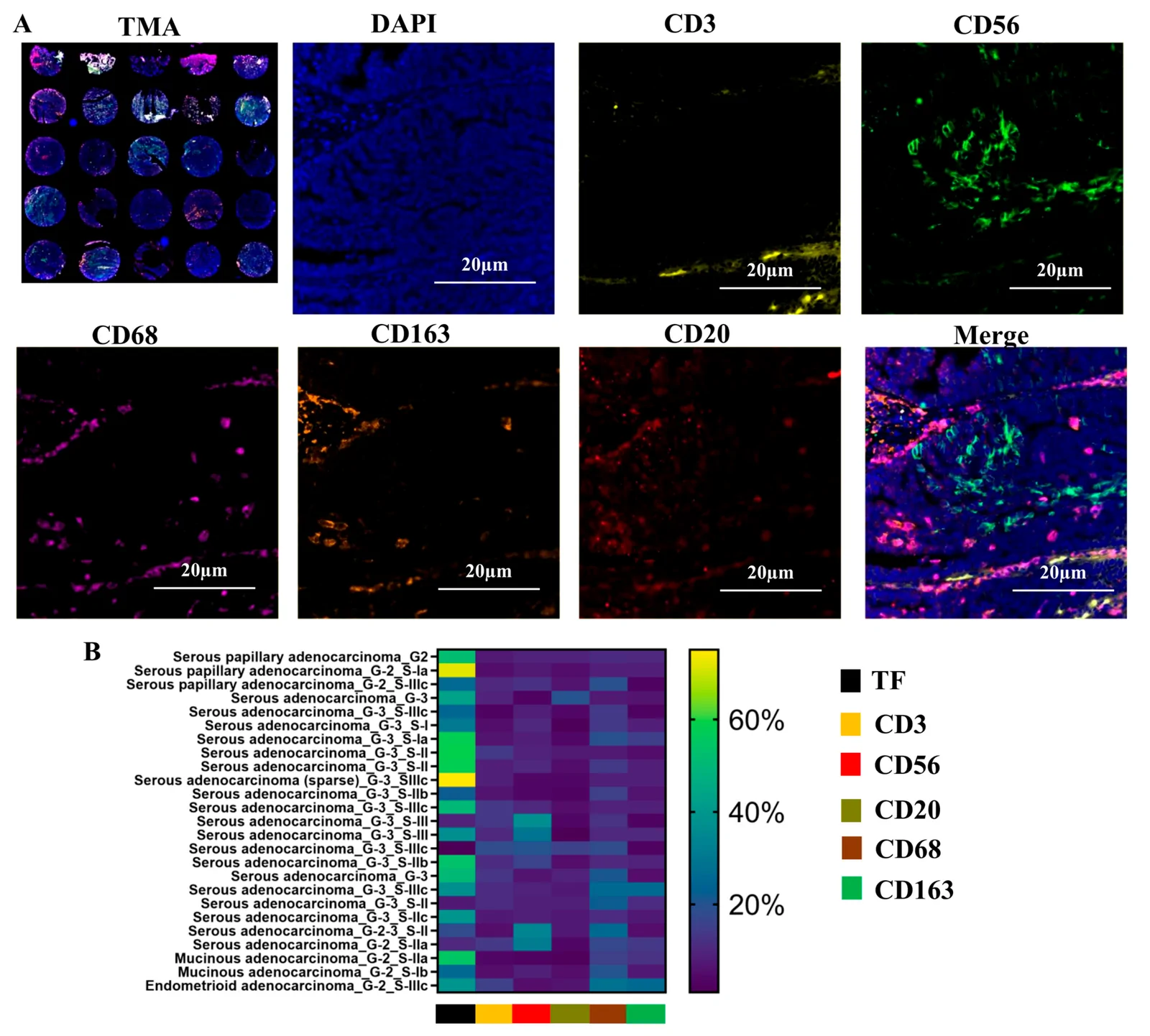
Multiplex immunofluorescence profiling of tumor-infiltrating immune cell populations in the ovarian cancer tissue microarray. (**A**) Representative multiplex images showing DAPI, CD3, CD56, CD68, CD163, CD20, and merged channels. (**B**) Heatmap of tissue factor and immune cell marker positivity across tumor microarray cores. Scale bar: 20 µm.

Multiplex immunofluorescence was used to examine the distribution of TF and immune-cell markers across the ovarian cancer TMA (Figure 6). Representative images showed distinct patterns among the immune-cell populations: CD56+ NK formed discrete clusters, whereas CD3+ T cells and CD68+/CD163+ macrophage populations were frequently distributed along stromal regions. Merged-channel images demonstrated the spatial proximity of multiple immune-cell subsets within the tumor microenvironment but did not, by themselves, establish cellular colocalization. Heatmap analysis revealed marked intercase heterogeneity in TF and immune-marker positivity across histologic subtypes and grades. CD3+ T cells and CD163+ macrophages were generally more abundant than CD20+ B cells or CD56+ NK cells in most cores.

### 2.7. High TF Expression Is Associated with Reduced NK-Cell and CD68+ Macrophage Infiltration and a Lower CD68/CD163 Ratio in the Ovarian Cancer TMA

**Figure 7:**
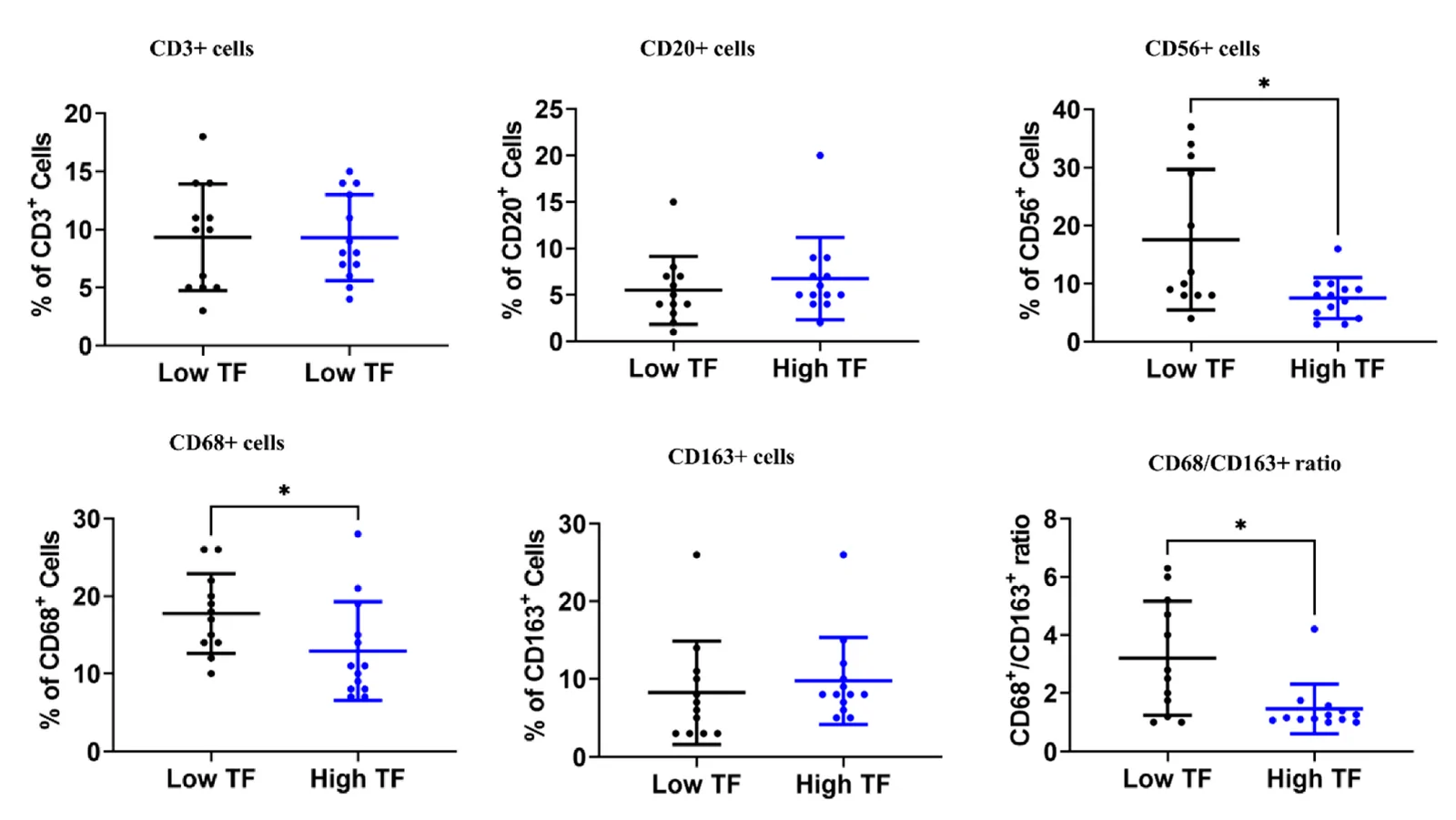
Association between TF expression and immune-cell infiltration in the ovarian cancer TMA. Proportions of CD3+, CD20+, CD56+, CD68+, and CD163+ cells, and the CD68/CD163 ratio were compared between low-TF and high-TF groups defined by the median TF-expression value. High TF tumors had lower CD56+ NK-cell and CD68+ macrophage infiltration and a lower CD68/CD163 ratio (two-tailed Mann-Whitney U test, *, *p* < 0.05). CD3+ T cells, CD20+ B cells, or CD163+ macrophage proportions did not differ significantly between groups (all *p* > 0.05).

To assess the relationship between TF expression and immune-cell infiltration in the TMA, cases were dichotomized at the median TF-expression value (Figure 7). High-TF tumors contained significantly lower proportions of CD56+ NK cells and CD68+ macrophages and had a significantly lower CD68/CD163 ratio than low-TF tumors. The proportions of CD3+ T cells, CD20+ B cells, and CD163+ macrophages did not differ significantly between groups, although CD163+ cells showed a nonsignificant tendency toward higher abundance in the high-TF group.

**Figure 8:**
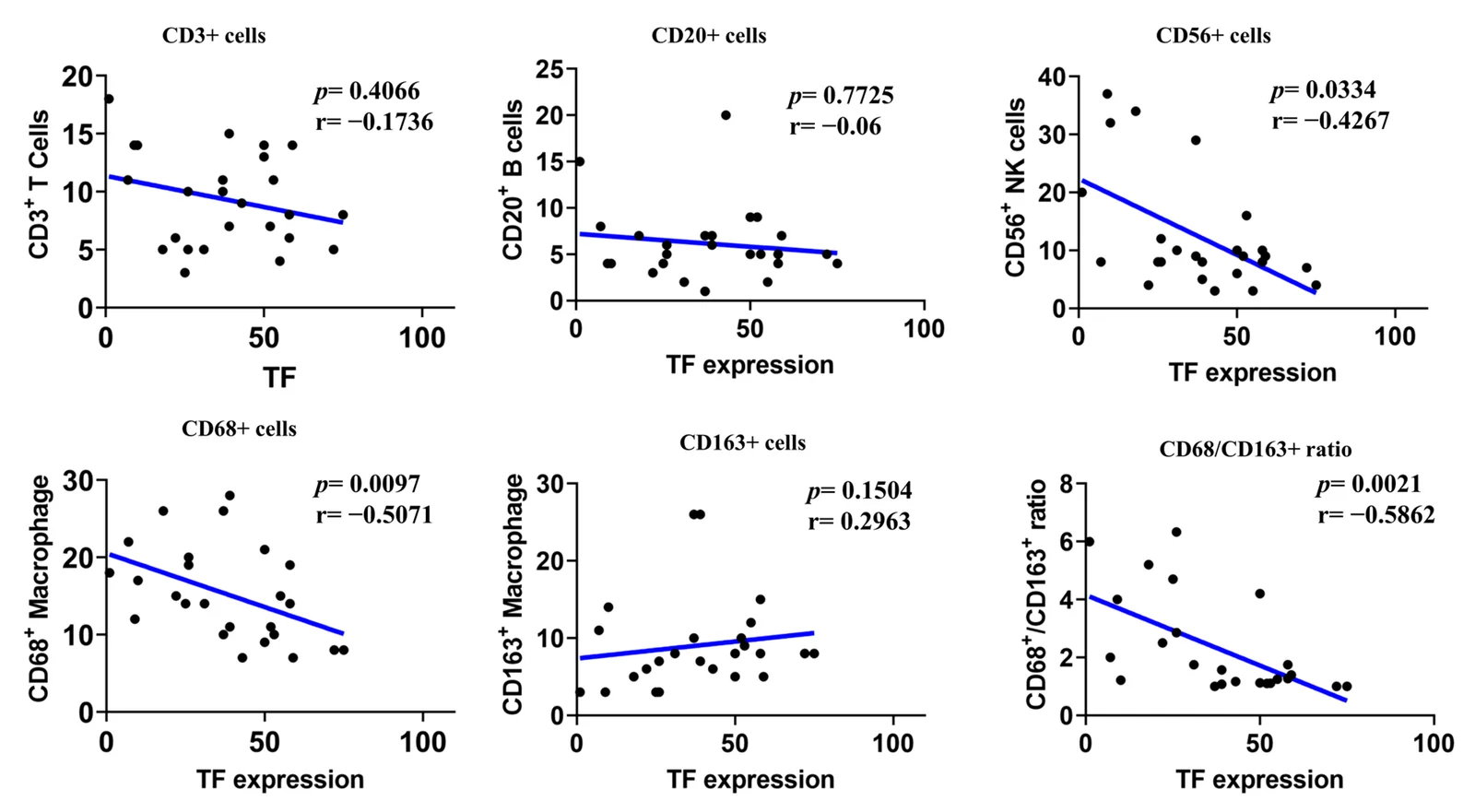
Correlations between TF expression and immune-cell markers in the ovarian cancer TMA. Scatter plots show the relationships between TF expression and CD3+, CD20+, CD56+, CD68+, and CD163+ cell abundance and the CD68/CD163 ratio.

Continuous-variable analyses were consistent with the group comparison. TF expression correlated negatively with CD56+ NK-cells abundance (r = −0.4267, *p* = 0.0334), CD68+ macrophage abundance (r = −0.5071, *p* = 0.0097), and the CD68/CD163 ratio (r = −0.5862, *p* = 0.0021) (Figure 8). TF expression was not significantly correlated with CD3+ T cells (r = −0.1736, *p* = 0.4066) or CD20+ B cells (r = −0.0600, *p* = 0.7725), and the positive correlation with CD163+ macrophages was not statistically significant (r = 0.2963, *p* = 0.1504). Overall, the TMA data associate higher TF expression with reduced NK-cell and CD68+ macrophage abundance and with a lower CD68/CD163 ratio, rather than with broad suppression of all immune-cell populations examined. The CD68/CD163 ratio finding suggests a relative shift toward CD163 positivity with the macrophage compartment but does not establish functional macrophage polarization. These comparisons were not corrected for multiple testing, and the results should be considered exploratory.

## 3. Discussion

This study integrated scRNA-seq, TCGA-based transcriptomics and immune-deconvolution analyses, and multiplex immunofluorescence in an independent TMA to characterize the relationship between TF expression and the ovarian tumor immune microenvironment. TF expression was concentrated in tumor epithelial cells and a discrete endothelial subpopulation and was not significantly associated with stage, grade, DFS, or OS. Nevertheless, TF-stratified survival analyses revealed different prognostic patterns for CD8+ T cell and M1 macrophage infiltration, and high-TF tumors had lower CD56+ NK-cell and CD68+ macrophage infiltration and a lower CD68/CD163 ratio. Collectively, these findings support an association between TF expression and less favorable immune context in ovarian cancer, while remaining insufficient to establish causality.

The concentration of TF expression in tumor epithelial cells and a discrete endothelial subcluster is broadly consistent with reports identifying TF as a tumor-cell–associated protein in several solid cancers, including ovarian cancer, with limited expression in infiltrating immune cells [11]. The functional role of TF in the endothelial subcluster was not tested directly and remains unclear. TF-high cells were also enriched for ribosome biogenesis, DNA replication, MHC class II antigen presentation, and RNA-splicing signatures. These associations may reflect TF-related cell states, but they may also be influenced by differences in cell-type composition; therefore, they should not be interpreted as TF-driven pathways without perturbation experiments and analyses that account for cellular identity.

TF expression was not significantly associated with DFS, OS, stage, or grade in the TCGA cohort. Because the survival analyses were unadjusted, these findings should not be interpreted as evidence that TF lacks independent prognostic value. Previous studies have linked TF to other clinically relevant endpoints, including venous thromboembolism in ovarian cancer, particularly in clear-cell histology [59]. Moreover, survival in ovarian cancer is strongly influenced by residual disease after surgery, treatment sensitivity, histologic and molecular subtype, and other covariates that were not incorporated into the present analyses. TF may therefore have limited prognostic value as a single marker while still identifying a biologically distinct immune context. Multivariable models in larger, clinically annotated cohorts will be required to evaluate this possibility.

One of the most notable findings was the different association between CD8+ T cell infiltration and survival across TF strata. CD8+ T cell infiltration, particularly within the intraepithelial compartment, is generally associated with favorable outcomes in ovarian cancer [60], a pattern supported by subsequent meta-analyses [61]. In the present analysis, high CD8+ T cell infiltration was associated with better survival in the low-TF subgroup (HR = 0.6) but with worse survival in the high-TF subgroup (HR = 1.72). High M1 macrophage infiltration was likewise favorable only in the low-TF subgroup. These results raise the possibility that TF expression marks an immune context in which infiltrating effector cells are less functional or are accompanied by another adverse feature. Preclinical studies have linked TF-FVIIa and TF-FVIIa-FXa signaling through PAR2 immunomodulatory cytokine production and macrophage-mediated suppression of cytotoxic T cells function [17,62,63], providing a plausible biological framework. However, our data do not measure T cell function, and apparent differences between TF strata were derived from exploratory subgroup analyses without a formal interaction test. Because multiple immune-cell subsets were examined, these findings require validation in independent cohorts.

The lower CD56+ NK-cell and CD68+ macrophage infiltration, and lower CD68/CD163 ratio in high-TF tumors are broadly consistent with reports associating a high CD163/CD68 ratio with adverse outcomes in ovarian cancer [64,65]. However, CD68 and CD163 are not exclusive functional markers, and their ratio cannot by itself establish M1/M2 polarization. The absence of a significant difference in CD163+ cell abundance also argues against overinterpretation, particularly given the small TMA cohort. Reduced NK-cell infiltration is compatible with mechanisms by which coagulation-pathway activation, including platelet activation and platelet-derived TGF-β, can impair NK-cell receptor expression and cytotoxicity [66,67,68]. We present this mechanism only as a hypothesis because platelet activation, TGF-β signaling, and NK-cell function were not measured in our samples. TF expression also correlated positively with checkpoint gene expression, most strongly with TIM-3 (HAVCR2). However, TIM-3 is not T cell-restricted and is also expressed on macrophages and NK cells, so this correlation may partly reflect myeloid composition rather than T cell dysfunction specifically.

These observations may have some translational relevance because TF is already therapeutically targetable. Tisotumab vedotin, a TF-directed antibody-drug conjugate, is approved for cervical cancer and is being investigated in other TF-expressing tumors, including ovarian cancer [69,70]. Additional TF-targeting approaches, including near-infrared photoimmunotherapy and multi-antigen antibody-drug conjugate platforms, have also been explored in ovarian and other solid tumors [12,13,14,15]. The present data, however, do not demonstrate that TF targeting will restore antitumor immunity or improve clinical outcomes in ovarian cancer. Functional studies and biomarker-driven clinical analysis are needed to determine whether TF expression can identify patients most likely to benefit from TF-directed therapy, alone or in rational immune-based combinations.

The TCGA and TMA immune findings should not be treated as directly convergent, because the two platforms measure different things. CIBERSORT’s CD8+ T cell, activated NK-cell, and M1 macrophage estimates are transcriptomic signatures based on cell function. The TMA panel used simpler lineage markers (CD3, CD56, CD68/CD163) that cannot separate CD8+ from CD4+ T cells, resting from activated NK cells, or M1 from M2 macrophages. This likely explains why the two platforms disagreed: the negative TCGA correlation between TF and CD8+ T cells did not appear for CD3+ T cells in the TMA; the null TCGA result for NK cells contrasted with a significant negative TMA result for CD56+ cells; and the TCGA M1 correlation contrasted with a negative CD68+ finding and a non-significant positive CD163+ trend in the TMA. These differences most likely reflect marker specificity, not opposing biology. Still, the two cohorts should be seen as partially overlapping, not mutually confirming.

Several limitations should be considered. First, CIBERSORT estimates from bulk RNA-sequencing data are computational inferences rather than direct measurements and may be less reliable for low-abundance populations [71,72]. Second, the TMA cohort was small (n = 25), including multiple histologic subtypes, and lacked stage information for three cases, limiting statistical power and the reliability of stage- and grade-stratified analysis. Median dichotomization of TF and immune-cell estimates also reduced continuous information and may have produced threshold-dependent subgroup effects. Third, the survival analysis was not adjusted for major clinical covariates, no formal interaction test was performed, and multiple immune-cell populations were evaluated; therefore, the subgroup findings should be considered exploratory. Fourth, the single-cell dataset represented a limited number of tumors at a single time point and may not capture interpatient, treatment-related, or temporal variation. Fifth, marker-based TMA analyses cannot fully resolve immune-cell functional states, and the CD68/CD163 ratio is not a direct measure of macrophages. Sixth, the multiplex TMA panel included a pan-T cell marker (CD3) rather than CD8, so the protein-level cohort does not directly test the CD8+ T cell-specific survival association identified in the TCGA analysis. Seventh, the differential hazard ratios for CD8+ T cell infiltration across TF strata were compared descriptively rather than with a formal statistical interaction test, so this should be regarded as a suggestive, hypothesis-generating pattern rather than demonstrated effect modification. Finally, the study was correlative and did not test whether TF perturbation directly alters CD8+ T cell function, macrophage state, or NK-cell activity. The proposed mechanisms are therefore hypothesis-generating and require experimental validation.

## 4. Materials and Methods

### 4.1. Single-Cell Transcriptomic Analysis

Single-cell RNA-sequencing (scRNA-seq) data from the ovarian cancer dataset GSE232314 were obtained from the Gene Expression Omnibus (GEO) and analyzed in R using Seurat v5.0. The dataset comprised 10x Genomics single-cell RNA-seq data from 2 patients (4 samples total, representing CD45-negative and CD45-positive flow-sorted fractions per patient), sequenced on an Illumina NovaSeq 6000 (GPL24676, Illumina, Inc., San Diego, CA, USA). Tissue was derived from high-grade ovarian serous carcinoma. The dataset initially contained 23,408 cells. Cells were removed if they had 200 or fewer detected genes, 500 or fewer UMI counts, or 20% or more mitochondrial reads, which excluded 3303 cells and left 20,105. Doublets were identified separately for each sample with DoubletFinder v2.0.6, using an expected doublet rate of 0.8% per 1000 cells adjusted for homotypic doublets, with the pK parameter selected by parameter sweep on a random subsample of up to 2000 cells per sample. This removed 691 putative doublets, leaving 19,414 cells for downstream analysis. Data were log-normalized, highly variable genes were identified, and the data were scaled and subjected to principal component analysis. The first 20 principal components were used for graph-based clustering and UMAP visualization. Clusters were annotated using canonical marker genes as tumor epithelial cells, fibroblasts, endothelial cells, macrophages, T cells, B cells, NK cells, cancer-associated fibroblasts, mesothelial cells, dendritic cells, plasma cells, cycling tumor cells, or other myeloid cells. TF expression was visualized across the annotated UMAP clusters to identify the cell populations in which it was concentrated. Feature plots for *CD3D/CD3E* (T cells), *NCAM1* (NK cells), *MS4A1* (B cells), *CD68* (macrophages), and *CD163* (CD163-expressing macrophages) were generated to compare their cluster-level transcriptomic distributions with that of TF. The frequency and distribution of TF-expressing cells across annotated cell types were quantified to identify the predominant cellular sources of TF expression.

### 4.2. Pathway and Functional Enrichment Analysis

To characterize the transcriptional programs associated with TF expression, differentially expressed genes were identified between TF-high and TF-low cell populations using Seurat’s Find Markers function (Wilcoxon rank-sum test; adjusted *p* < 0.05; |log2 fold change| ≥ 0.25). Gene Ontology (GO) Biological Process enrichment analysis was performed on the resulting gene set to identify overrepresented functional categories. Gene set enrichment analysis (GSEA) was performed using a pre-ranked gene list based on log2 fold-change values to identify enriched Hallmark and canonical pathways. Enrichment analyses were conducted in R using clusterProfiler v4.18.4, and statistical significance was defined as Benjamini-Hochberg-adjusted *p*-value < 0.05.

### 4.3. TCGA Ovarian Cancer Data Analysis

Transcriptomic and clinical data for ovarian cancer patients were obtained from The Cancer Genome Atlas (TCGA). TF expression across tumor stages and histologic grades was analyzed using the UALCAN web portal (https://ualcan.path.uab.edu/), which reports normalized transcripts-per-million (TPM) values derived from TCGA RNA-sequencing data. Clinicopathologic subgroup comparisons were generated using UALCAN’s built-in statistical analysis tools. UALCAN’s clinicopathologic comparisons used (n = 302) TCGA-OV cases with a UALCAN-computed TPM median split. For survival analysis, the GEPIA2 platform (http://gepia2.cancer-pku.cn/) was used to dichotomize patients into low- and high-TF groups at the median expression values. Kaplan–Meier curves were generated for disease-free survival (DFS) and overall survival (OS), and group differences were assessed using the log-rank test. Hazard ratios (HRs) were reported for the high-TF group relative to the low-TF group. Patients were dichotomized into low- and high-TF expression groups using a median split (cutoff-high 50%, cutoff-low 50%), based on *TF* expression alone. Kaplan–Meier curves were generated with axis units in months, and group differences were assessed using the log-rank test. Hazard ratios (HRs) were calculated for the high-TF group (n = 212) relative to the low-TF group (n = 212).

### 4.4. Immune Infiltration Analysis via TIMER2.0/CIBERSORT

To examine whether the prognostic association of immune infiltration differed according to TF expression, immune-cell abundance was estimated by CIBERSORT deconvolution through the TIMER2.0 web platform (http://timer.cistrome.org/) applied to the TCGA-OV cohort (n = 303). For each immune cell population of interest—naïve B cells, memory B cells, CD8^+^ T cells, activated NK cells, and M1 macrophages and M2 macrophages—patients were categorized into four groups defined by median-dichotomized TF expression and median-dichotomized immune-cell abundance: low TF/low infiltration, low TF/high infiltration, high TF/low infiltration, and high TF/high infiltration. Kaplan–Meier curves were generated for the four groups. Within each TF stratum, high versus low immune infiltration was compared using the log-rank test, and HRs with corresponding *p*-values were reported.

To assess the association between TF expression and immune-cell abundance, TIMER2.0 CIBERSORT estimates based on the LM22 signature matrix were analyzed for the TCGA ovarian cancer cohort (n = 303). Spearman’s rank correlation analysis was used to relate TF expression to the estimated infiltration fractions of naïve B cells, memory B cells, CD8^+^ T cells, activated NK cells, M1 macrophages, and M2 macrophages. Spearman correlation coefficients and *p*-values were reported, with *p* < 0.05 considered statistically significant. Because multiple immune-cell subsets were examined, these correlation analyses were considered exploratory. Correlations between TF and the immune checkpoint genes CD274 (PD-L1), CTLA4, and HAVCR2 (TIM-3) were assessed in the TCGA ovarian cancer (OV) cohort using the GEPIA2 correlation analysis tool (http://gepia2.cancer-pku.cn/), with gene expression reported as log2 (TPM) and correlation strength expressed as the Spearman correlation coefficient (R).

### 4.5. Multiplex Immunofluorescence Analysis of an Ovarian Cancer Tissue Microarray (TMA)

An ovarian cancer tissue microarray (TMA; OV1003, TissueArray.com, Derwood, MD, USA) containing 25 tumor samples was used for independent protein-level assessment. Available clinicopathologic information, including tumor stage and grade, was obtained from the vendor. TF expression was quantified in individual TMA cores and compared across stage and grade groups.

The multiplex immunofluorescence staining was performed by following the protocol of Sheng et al. 2026 [73]. TMA sections were incubated at 60 °C for 40 min and deparaffinized in xylene for 20 min. Sections were then rehydrated through graded ethanol (100%, 2 × 5 min; 90%, 5 min; 80%, 5 min; and 70%, 5 min) and rinsed twice in deionized water. Antigen retrieval was performed in sodium citrate buffer (pH 6.0) using microwave heating for 20 min. Endogenous peroxidase activity was blocked with 3% hydrogen peroxide for 10 min, after which sections were washed three times with water and blocked with 10% goat serum in phosphate-buffered saline (PBS) for 1 h. Sections were incubated overnight at 4 °C in a humidified chamber with an anti-TF antibody (CD142 mAb, clone HTF-1; Miltenyi Biotec, Bergisch Gladbach, Germany, catalog no. 130-098-741; 10 μL). The next day, slides were equilibrated to room temperature for 1 h, washed three times with PBST (PBS containing 0.1% Tween 20), and incubated for 1 h with horseradish peroxidase (HRP)-conjugated anti-mouse IgG (Invitrogen, Waltham, MA, USA, catalog no. G21040). After three PBST washes, signal was developed with iFluor 430 tyramide (AAT Bioquest, Pleasanton, CA, USA, catalog no. 45096) for 10 min, followed by three additional PBST washes. Slides were mounted in DAPI-containing mounting medium (Vector Laboratories, Newark, CA, USA, catalog no. H-1200) for fluorescence imaging.

The same region of TMA sections was also used for immune marker staining using the same protocols described above. The sections were incubated overnight at 4 °C in a humidified chamber with primary anti-CD3 antibody (Invitrogen, #14-0030-85, 1:100), anti-CD56 antibody (Invitrogen, #MA5-11563, 1:100), anti-CD20 antibody (Invitrogen, #14-0202-82, 5 µg/mL), anti-CD163 (Sigma-Aldrich, St. Louis, MO, USA, #HPA046404-100UL, 1:500) antibody and anti-CD68 (NeoBiotechnologies, Union City, CA, USA, #968-MSM2-P1, 1 µg/mL) antibody. Appropriate anti-mouse or anti-rabbit secondary antibodies were then applied according to the host species of the primary antibody. Signal amplification was carried out using iFluor 430 Tyramide (AAT Bioquest, Pleasanton, CA, USA, #45096), Alexa Fluor 488 Tyramide (Invitrogen, #B40953), Alexa Fluor 546 Tyramide (Invitrogen, #B40954), Alexa Fluor 647 Tyramide (Invitrogen, #B40958), and iFluor 750 Tyramide (AAT Bioquest, #45065) following the same staining protocol described above. Finally, slides were mounted using DAPI-containing mounting medium (Vector Laboratories, #H-1200) for fluorescence imaging. Fluorescence imaging was conducted using the Axioscan 7 slide scanner (Zeiss, Oberkochen, Germany) with ZEN imaging software (ZEN 3.4). Quantitative image analysis was performed using the open-source digital pathology software QuPath (v0.5.1) [74].

### 4.6. Analysis of Immune Cell Infiltration According to TF Expression

Patients within the TMA cohort were stratified into low- and high-TF expression groups based on the median TF expression value. Comparative analyses of immune cell infiltration were performed for CD3+, CD56+, CD20+, CD68+, and CD163+ cell populations between the two groups to determine the association between TF expression and immune infiltration within the ovarian tumor microenvironment. In addition, correlation analyses were performed treating TF expression as a continuous variable to assess its relationship with CD3+, CD56+, CD20+, CD68+, and CD163+ cell populations, using correlation coefficients and corresponding statistical significance testing.

### 4.7. Statistical Analysis

All statistical analyses were performed using GraphPad Prism 9.0.0 (GraphPad Software, San Diego, CA, USA) and R v4.5.1. Data are presented as mean ± standard deviation unless otherwise indicated. Comparisons between groups were performed using appropriate statistical tests. Kaplan–Meier survival analysis was conducted using the log-rank test, and *p*-values < 0.05 were considered statistically significant.

## 5. Conclusions

In conclusion, TF expression in ovarian cancer was concentrated in tumor epithelial cells and a discrete endothelial subpopulation. Although TF expression alone was not significantly associated with stage, grade, DFS, or OS, it was associated with lower estimated CD8+ T cell, memory B cell, and M1 macrophage infiltration, and with TF-stratum-specific prognostic patterns for CD8+ T cells and M1 macrophages. Independent TMA analysis further associated high TF expression with lower CD56+ NK-cell and CD68+ macrophage infiltration and a lower CD68/CD163 ratio. These findings, while not fully concordant in their specifics across the transcriptomic and protein-level cohorts, together support TF as a candidate marker of the tumor immune context. Prospective validation, formal interaction analyses, and mechanistic studies are needed before TF can be established as an immune-related biomarker in ovarian cancer.

## Figures and Tables

**Table 1:** Clinicopathologic characteristics of the ovarian cancer TMA cohort. Stage information was unavailable for three cases.

Characteristic	Category	n
Sex	Female	25
Age (years)	Median (range)	51 (39–66)
Stage	I	3
	II	9
	III	10
Grade	2	8
	3	17
Histology	Serous papillary adenocarcinoma	3
	Serous adenocarcinoma	19
	Mucinous adenocarcinoma	2
	Endometrioid adenocarcinoma	1

## Data Availability

The original contributions presented in this study are included in the article. Further inquiries can be directed to the corresponding author.

## Supplementary Materials

The following supporting information can be downloaded at: https://www.mdpi.com/article/10.3390/ijms27209148/s1.
