# Supplementary material for: Tissue Factor Expression Is Associated with Distinct Pattern of Immune-Cell Infiltration in the Ovarian Tumor Microenvironment
Source: Int J Mol Sci. 2026 Oct 15;27(20):9148. doi: 10.3390/ijms27209148 (PMC13705184; doi:10.3390/ijms27209148)
Supplement: Supplementary file 1 [file ijms-27-09148-s001.zip › ijms-4595501-supplementary.pdf]

Supplimentary Figure

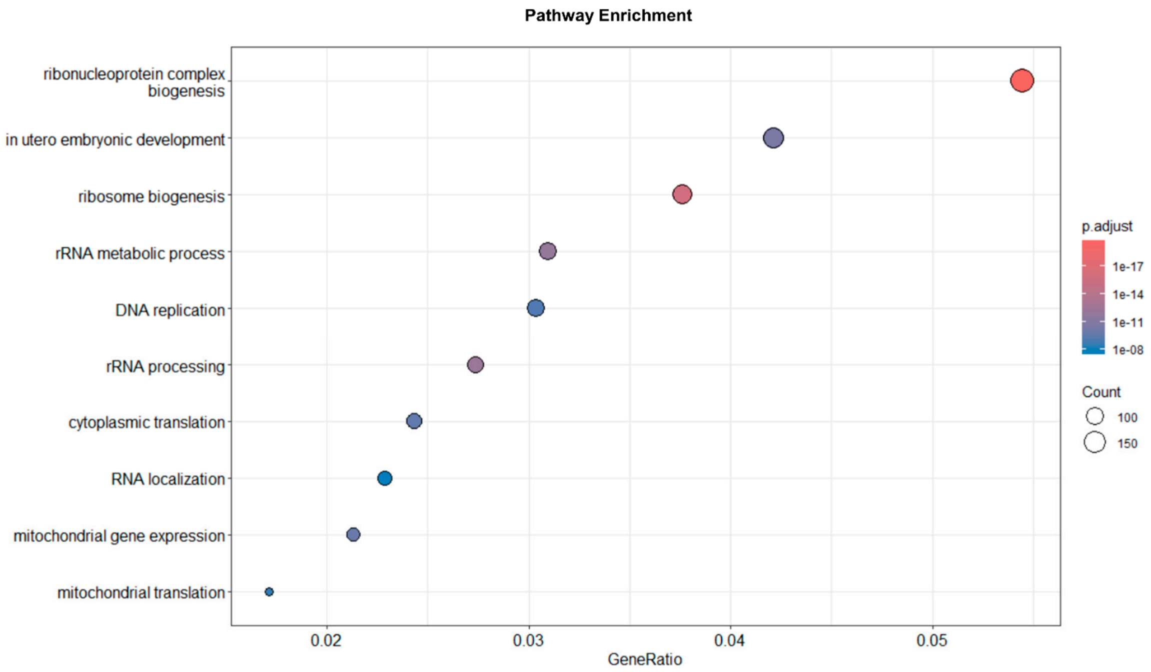

Figure S1: Gene Ontology biological process enrichment analysis of TF-associated differentially expressed genes (Wilcoxon rank-sum test, adjusted $p < 0.05$), showing significant enrichment for ribonucleoprotein complex biogenesis, ribosome biogenesis, rRNA metabolic processing, and DNA replication.

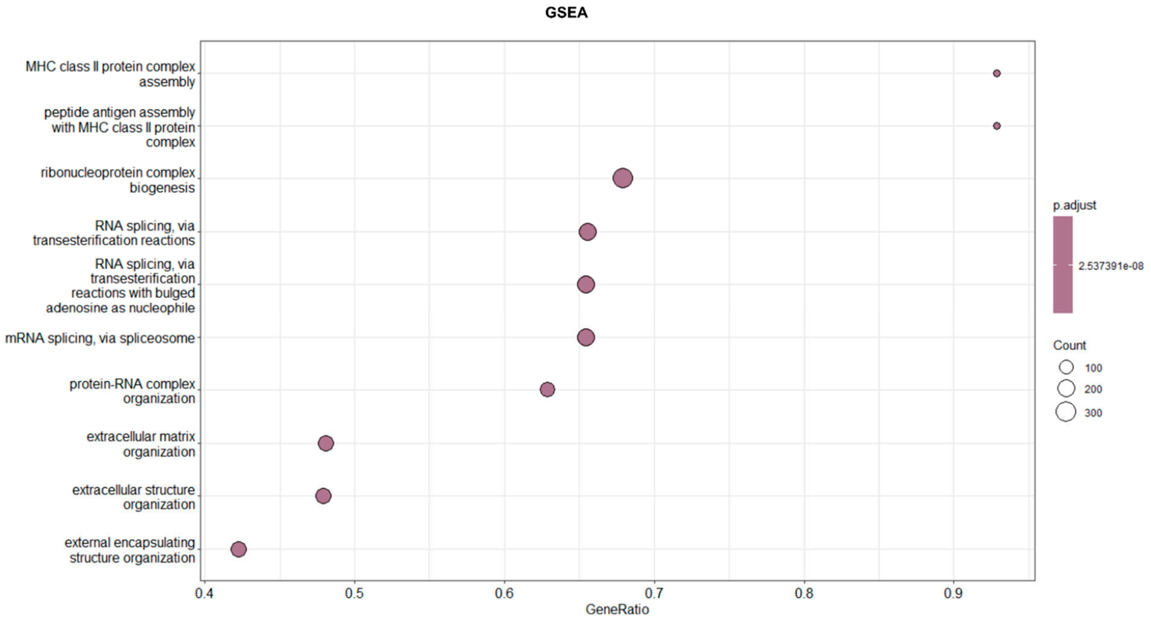

Figure S2: GSEA of TF-high versus TF-low cells, showing significant enrichment of MHC class II antigen presentation and RNA splicing pathways (FDR-adjusted $p < 0.05$).

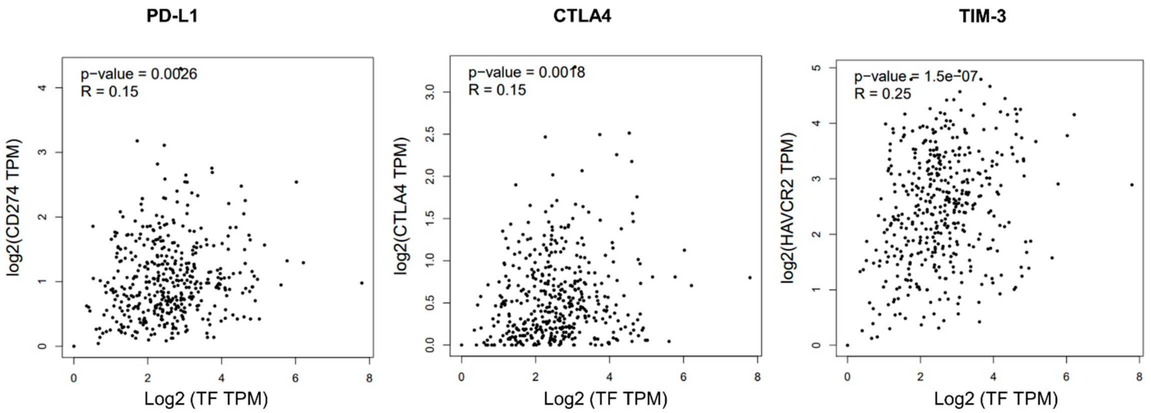

Figure S3: Correlation of TF expression with immune checkpoint markers. Scatter plots showing the correlation between TF expression and immune checkpoint marker expression in TCGA cohort using GEPIA2. TF expression was positively correlated with PD-L1 (CD274) expression, CTLA4 expression , and TIM-3 (HAVCR2) expression. Gene expression values are presented as $\log_2$-transformed transcripts per million (TPM).
